# Facile Enzymatic Synthesis of Base J-Containing Oligodeoxyribonucleotides and an Analysis of the Impact of Base J on DNA Replication in Cells

**DOI:** 10.1371/journal.pone.0103335

**Published:** 2014-07-25

**Authors:** Debin Ji, Yinsheng Wang

**Affiliations:** Department of Chemistry, University of California Riverside, Riverside, California, United States of America; Tulane University Health Sciences Center, United States of America

## Abstract

We reported here the use of T4 bacteriophage β-glucosyltransferase (T4 β-GT) for the facile synthesis of base J-containing oligodeoxyribonucleotides (ODNs). We found that the enzyme could catalyze the glucosylation of 5-hydroxymethyl-2-deoxyuridine (5hmU) in both single- and double-stranded ODNs, though the latter reaction occurred only when 5hmU was mispaired with a guanine. In addition, base J blocked moderately DNA replication, but it did not induce mutations during replication in human cells.

## Introduction

**Figure 1:**
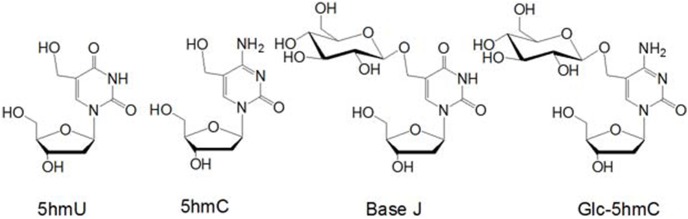
Chemical Structures of 2′-deoxynucleosides containing 5hmU, 5hmC, base J and Glc-5hmC.

Unicellular eukaryotic kinetoplastid flagellates, such as *Trypanosoma* and *Leishmania* species, contain a unique modified base, 5-(β-D-glucosylhydroxymethyl)uracil (a.k.a. base J), in their nuclear DNA (Figure 1) [1], [2]. Base J replaces approximately 1% of thymine (T) in the nuclear DNA of these species, but this modified base is absent in other eukaryotes, prokaryotes or viruses, rendering biosynthesis of J a potential therapeutic target against pathogenic kinetoplastids [3]. Base J is produced by oxidation of thymine to 5-hydroxymethyluracil and subsequent glucosylation of the latter modified nucleobase [3]. The oxidation step is catalyzed by the J-binding proteins JBP1 and JBP2, which are members of the TET-JBP superfamily of dioxygenases [4], [5]. Along this line, the mammalian TET enzymes catalyze the corresponding oxidation of 5-methylcytosine to 5-hydroxymethylcytosine [6] as well as further oxidation of the latter to 5-formylcytosine and 5-carboxylcytosine [7], [8].

The enzyme catalyzing the glucose transfer reaction involved in base J biosynthesis has yet been identified, though many experimental approaches including complementation in cell extracts and RNAi knockdown of candidate genes have been attempted [4]. In this vein, it is worth noting that, through bioinformatic analysis of biochemical pathways for DNA modifications, Avarind et al. [9] recently identified a putative glucosyltransferase with an operonic association to a JBP-related gene in several phage genomes. The authors postulated that the TET/JBP-associated glycosyltransferases (or TAGTs) may glycosylate substrates (i.e., 5-hydroxymethyluracil) generated by the JBP-like enzymes [9]. Interestingly, the bioinformatic analysis also revealed the presence of the mammalian ortholog of this glucosyltransferase (i.e., GREB1) [9], raising the possibility of the existence of J analog in mammalian genome.

In *Leishmania*, a small fraction of base J is located at transcription termination sites [10]. Loss of base J results in massive transcription read-through at these sites, suggesting that base J regulates RNA polymerase II (Pol II)-mediated transcription by stalling Pol II or specifying transcriptional termination [10]. It remains elusive whether base J affects other aspects of DNA metabolism, especially DNA replication.

Understanding the biological function and characterizing the biophysical properties of base J at the molecular level necessitate the availability of oligodeoxyribonucleotides (ODNs) containing a site-specifically inserted base J. Along this line, base J-carrying ODNs have been previously synthesized using conventional phosphoramidite chemistry and automated solid-phase DNA synthesis [11]–[13]. This method generally involves multiple synthetic steps, and it has relatively low overall yields. Several efficient chemoenzymatic approaches have been developed for the preparation of sugar-nucleotide derivatives [14]–[16]. We reason that a similar method might be useful for the synthesis of base J-containing ODNs. However, so far, enzymes that can efficiently catalyze this reaction have not been discovered yet. Here, we found that β-glucosyltransferase of *Escherichia coli* T4 bacteriophage (T4 β-GT) could catalyze the glucosylation of 5hmU in single-stranded ODN or in double-stranded ODN when 5hmU is mispaired with a guanine (G). Additionally, with the use of this method, we were able to prepare sufficient base J-containing ODN for assessing how base J compromises DNA replication in cells. We found that, in contrast to its strong blocking effects on DNA transcription, base J moderately impedes DNA replication in human cells and it does not induce mutations during this process.

## Materials and Methods

### Materials

Unmodified oligodeoxyribonucleotides (ODNs) used in this study were purchased from Integrated DNA Technologies (Coralville, IA, USA). [γ-^32^P]ATP was obtained from Perkin Elmer (Piscataway, NJ, USA). Shrimp alkaline phosphatase was obtained from the USB Corporation (Cleveland, OH, USA). T4 phage β-glucosyltransferase (T4 β-GT) and all other enzymes unless otherwise specified were purchased from New England BioLabs (NEB). 1,1,1,3,3,3-Hexafluoro-2-propanol (HFIP) was purchased from TCI America (Portland, OR, USA). Chemicals unless otherwise noted were obtained from Sigma-Aldrich (St. Louis, MO, USA).

The 5hmU-containing ODN (5′-ATGGCG**5hmU**GCTAT-3′) was synthesized following previously published procedures [17], and the identity of the modified ODN was confirmed by electrospray ionization-mass spectrometry (ESI-MS) and tandem MS (MS/MS) analyses [18]. We chose this particular sequence context because we previously conducted replication studies for a number of DNA lesions in the same sequence context [19]–[21].

### Detection of the T4 β-GT activity on different ODN substrates

The above 12 mer 5hmU-bearing ODN was annealed with a 20 mer complementary ODN (5′-ATAGCXCGCCATGAGCTCGAGA-3′) (‘X’ is an A, T, C or G). The single-stranded 12 mer 5hmU-bearing ODN or the annealed double-stranded ODNs (30 pmol each) were added to a 10-L T4 β-GT reaction buffer containing T4 β-GT (5 units) and uridine diphosphate-glucose (UDP-Glc, 0.04 mM). The mixture was incubated at 25°C for 30 min followed by heating at 65°C for 10 min. The above mixture (1 µL) was then incubated in a 10-L T4 polynucleotide kinase (T4 PNK) buffer with 5 mM DTT, ATP (50 pmol cold, premixed with 1.66 pmol [γ-^32^P]ATP) and 5 units of T4 PNK. The reaction was continued at 37°C for 1 h, followed by quenching with 10 µL formamide gel loading buffer containing xylene cyanol FF and bromophenol blue dyes. The mixture was loaded onto a 30% denaturing polyacrylamide gel (acrylamide:bis-acrylamide = 19∶1) containing 8 M urea (Figure S1 in File S1).

### Preparation of base J-containing ODN

**Figure 2:**
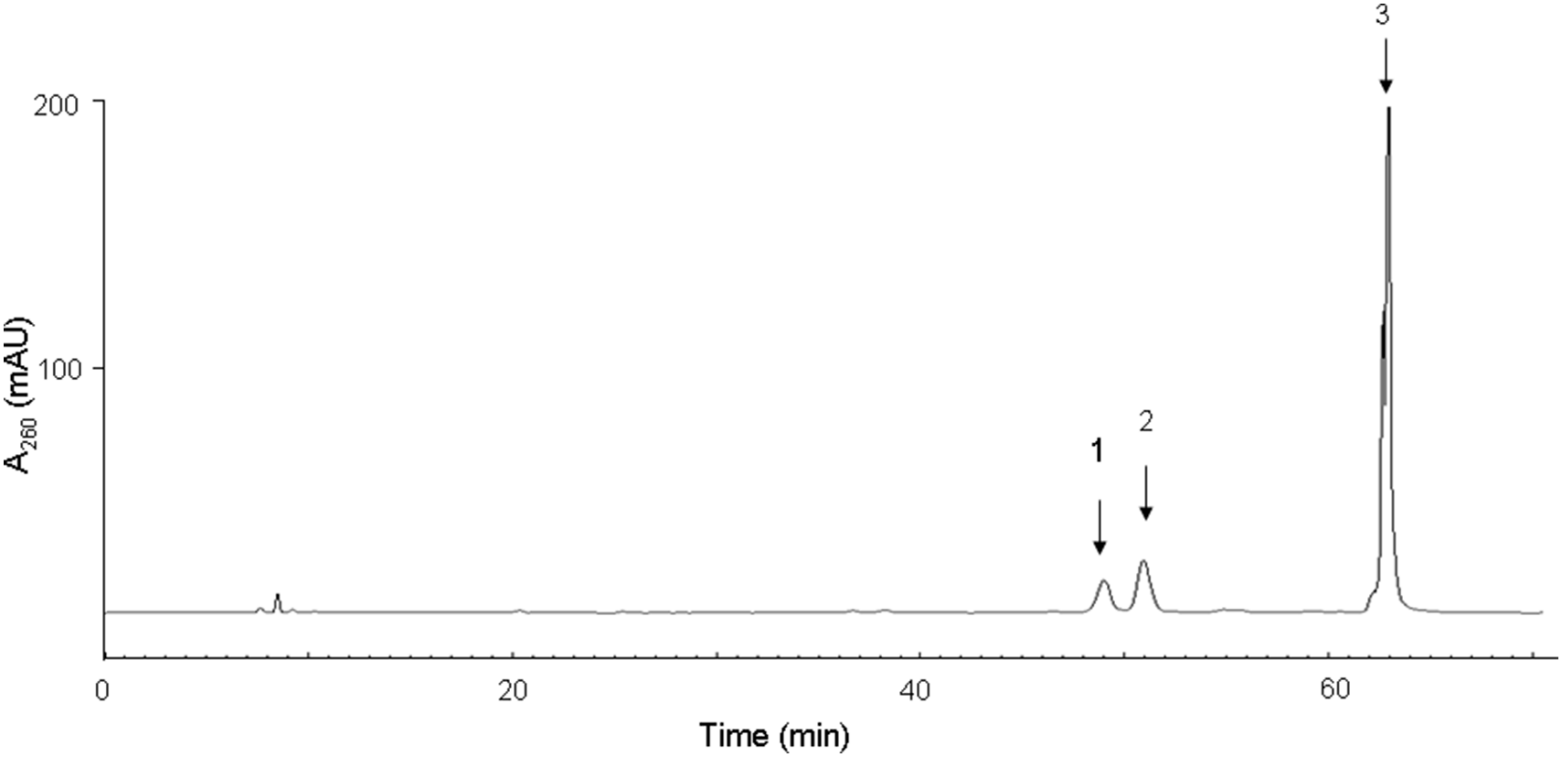
HPLC trace for the separation of a reaction mixture containing 12 mer base J- and 5hmU-containing ODNs. Peaks 1, 2 and 3 represent the 12 mer base J- and 5hmU-containing ODNs and complementary ODN, respectively.

**Figure 3:**
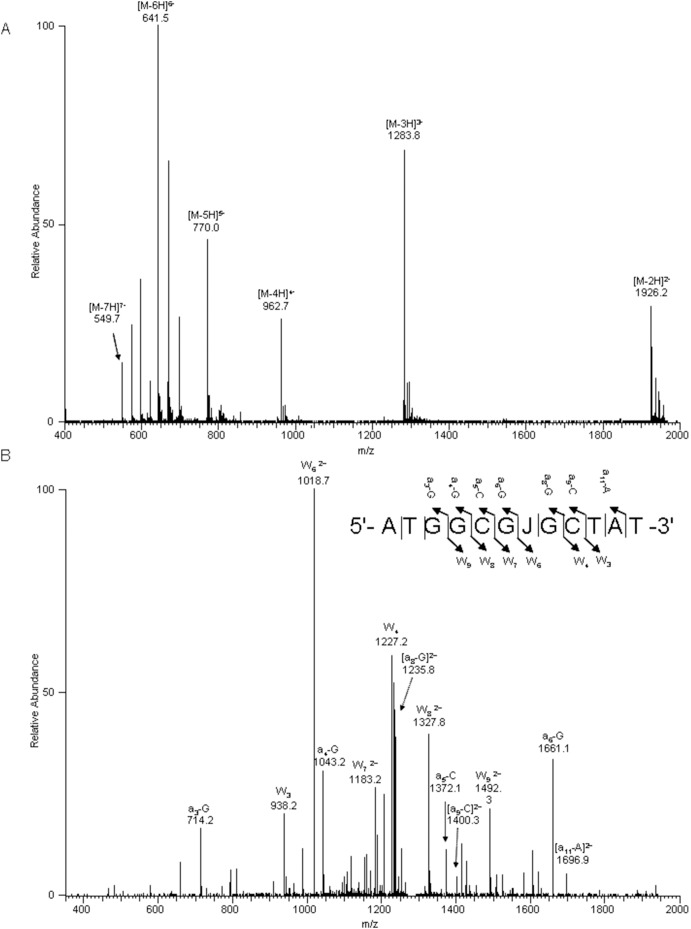
ESI-MS and MS/MS characterizations of d(ATGGCGJGCTAT) (‘J’ represents base J): (A) Negative-ion ESI-MS; (B) The product-ion spectrum of the [M–3H]^3–^ ion (*m/z* 1283.8).

For the preparation of base J-harboring ODN at a larger scale, 30 nmol of the 5hmU:G mismatch-containing double-stranded ODN, which was dissolved in a 1-mL NEB buffer 4, was mixed with a 50-L solution containing 2 mM UDP-Glc and 500 units T4 β-GT. After incubation at 37°C for 2 h, 200 units T4 β-GT and 10 µL 2 mM UDP-Glc were added, and the reaction was allowed to proceed at 37°C for up to 6 h. The reaction mixture was extracted once with phenol/chloroform/isoamyl alcohol (25∶24∶1, v/v). The aqueous portion was concentrated with Speed-vac, and separated by HPLC. The products were purified on a Beckman HPLC system with pump module 125 and a UV detector (module 126). A 4.6×250 mm Apollo C18 column (5 µm in particle size and 300 Å in pore size; Alltech Associate Inc., Deerfield, IL) was used. HFIP buffer (400 mM, pH adjusted to 7.0 with triethylamine, solution A) and methanol (solution B) were employed as mobile phases. The flow rate was 0.8 mL/min, and a gradient of 5–25% B in 5 min, 25–40% B in 50 min, and 40–80% B in 5 min was employed for the separation (Figure 2). The purified ODNs were desalted on the same HPLC system with H_2_O as mobile phase A and methanol as mobile phase B, and a gradient of 5% B in 20 min, 5–50% B in 1 min, and 50% B in 25 min was used. The identity of the base J-containing ODN was again confirmed by LC-MS/MS analysis (Figure 3), which was carried out using an Agilent 1100 capillary HPLC pump (Agilent Technologies, Palo Alto, CA) and an LTQ linear ion-trap mass spectrometer (Thermo Electron, San Jose, CA).

### Determination of bypass efficiency using competitive replication and adduct bypass assay

We first constructed the base J- and 5hmU-bearing double-stranded shuttle vector by using a previously described method (Figure S2 in File S1) [19], [22], [23]. Briefly, we nicked the parent vector with Nt.BstNBI to produce a gapped vector by removing a 25-mer single-stranded ODN, followed by filling the gap with a 12 mer base J- or 5hmU-bearing ODN (5′-ATGGCG**X**GCTAT-3′, X = base J or 5hmU), and a 13 mer unmodified ODN (5′-TGTGGAGTCGATG-3′). The resulting supercoiled lesion-bearing plasmid was isolated by agarose gel electrophoresis. Using the same method, we prepared the lesion-free control plasmid, where the 12 mer base J-containing ODN was replaced with a 12 mer ODN (5′-ATGGCG**T**GCTAT-3′) during the ligation reaction.

The HEK293T cells (6×10^5^) were seeded in 6-well plates and cultured overnight, after which they were transfected with 500 ng plasmids by using Lipofectamine 2000 (Invitrogen) following the manufacturer’s instructions. The cells were harvested at 24 h following transfection, and the progenies of the plasmid were isolated by using an alkali lysis method [24]. The residual unreplicated plasmids were removed by DpnI digestion, followed by digesting the resulting linear DNA with exonuclease III as described elsewhere [25].

The progeny plasmids were subsequently amplified by PCR using Phusion high-fidelity DNA polymerase (New England Biolabs, Ipswich, MA). The primers flanking the site where the lesion was initially inserted were 5′-CTTTCCAAAATGTCGTAACAACTCC-3′ and 5′-CAACACTCAACCCTATCTCGGTCTAT-3′, and the amplification cycle was 36, each consisting of 10 s at 98°C, 30 s at 65°C, and 45 s at 72°C, with a final extension at 72°C for 5 min. The PCR products were purified by QIAquick PCR Purification Kit (Qiagen) and stored at –20°C until use. For PAGE analysis, a portion of the PCR fragments was treated with 5 units NcoI and 1 unit shrimp alkaline phosphatase at 37°C in 10 µL NEB buffer 3 for 1 h, followed by heating at 80°C for 20 min to deactivate the shrimp alkaline phosphatase. The above mixture was then treated in a 15 µL NEB buffer 3 with 5 mM DTT, ATP (50 pmol cold, premixed with 1.66 pmol [γ-^32^P] ATP) and 5 units T4 PNK. The reaction was continued at 37°C for 1 h, followed by heating at 65°C for 20 min to deactivate the T4 PNK. To the reaction mixture was subsequently added 5 units SfaNI, and the solution was incubated at 37°C for 1 h, followed by quenching with 15 µL formamide gel loading buffer containing xylene cyanol FF and bromophenol blue dyes. The mixture was loaded onto a 30% polyacrylamide gel (acrylamide:bis-acrylamide = 19∶1) and products quantified by phosphorimager analysis. The bypass efficiency was calculated using the following formula, %bypass = (lesion vector bottom-strand signal/lesion vector top-strand signal)/(control vector bottom-strand signal/control vector top-strand signal).

### Identification of replication products by using LC-MS/MS

To further identify the replication products using LC-MS/MS, PCR products were treated with 50 units Nco I, 50 units SfaN I and 20 units shrimp alkaline phosphatase in 200-µL NEB buffer 3 at 37°C for 4 h, followed by heating at 65°C for 20 min (Figure S3 in File S1). The resulting solution was then extracted once with chloroform/isoamyl alcohol (24∶1, v/v), and the aqueous portion was desalted by HPLC and dried with Speed-vac and then dissolved in H_2_O. The ODN mixture was subjected to LC-MS/MS analysis on an LTQ linear ion-trap mass spectrometer, which was set up for monitoring the fragmentation of the [M–3H]^3−^ ions of the 13 mer ODNs [d(CATGGCGTGGTAT) and d(CATGGCGXGCTAT), where “X” designates A, T, C, or G].

## Results and Discussion

In nature, T4 β-GT catalyzes the transfer of a glucose residue from UDP-Glc to 5-hydroxymethyl-2′-deoxycytidine (5hmC) in double-stranded DNA, yielding 5-(β-glucosylhydroxymethyl)-2′-deoxycytidine (Glc-5hmC) (Figure 1) [26]. We reasoned that this enzyme may also be employed for the glucosylation of 5hmU to yield base J in DNA. To test this, we incubated a 12 mer ODN containing a single 5hmU with T4 β-GT in the presence of UDP-Glc. Our results showed that indeed T4 β-GT could catalyze the glucosylation of 5hmU in single-stranded ODN, albeit at a very low yield (Figure S1 in File S1). Hence, this result revealed that T4 β-GT possesses the enzymatic activity in inducing the glucosylation of 5hmU. T4 β-GT is known not to exhibit sequence specificity [26]; however, the crystal structure of T4 β-GT in complex with duplex DNA revealed the direct interaction between several amino acid residues in the protein with the G residue that is paired with 5hmC [27], [28]. This observation prompted us to ask whether the 5hmU residing in a 5hmU:G mispair may serve as a more robust substrate for T4 β-GT than when it is paired with an A. To exploit this possibility, we annealed the 12 mer 5hmU-containing ODN with 20 mer complementary sequences in which the nucleobase paired with 5hmU was an A, T, C or G. In this vein, we chose a 20 mer complementary strand because it can be resolved readily from the 5hmU- or base J-containing strand by PAGE or HPLC analysis (Figure 2). After treating these double-stranded ODNs with T4 β-GT, only the duplex with the 5hmU being mispaired with G gives the glucosylated product, with a yield being much higher than that in single-stranded ODN (Figure S1 in File S1, and the identity of the base J-containing ODN was confirmed by ESI-MS and MS/MS analyses, as depicted in Figure 3). The greater yield of base J obtained from duplex DNA substrate could be attributed to the stronger binding affinity of the enzyme toward double- than single-stranded DNA [29]. The higher reactivity observed for the 5hmU:G mispair than 5hmU:A, 5hmU:C or 5hmU:T base pair shows that the G in the opposite strand plays an instrumental role in binding with T4 β-GT and in facilitating the glucosylation of the opposing 5hmU.

Encouraged by the above results, we conducted the reaction at a larger scale by using 30 nmol of the above-described 5hmU:G mispair-containing 12 mer/20 mer duplex (See Materials and Methods), and subjected the reaction mixture to HPLC analysis. Our results showed that the 12 mer base J- and 5hmU-containing ODNs could be completely resolved from each other and from the complementary 20 mer ODN by HPLC (Figure 2). After HPLC separation and desalting, a total of 7.2 nmol base J-containing ODN was obtained at a yield of 24%. Furthermore, the unreacted 5hmU-containing ODN could be recovered for the next round of reaction. T4 β-GT does not display strong sequence specificity, and it can glucosylate all available 5-hmC bases in DNA [26], [30]; therefore, the method should be applicable for the synthesis of base J-containing ODNs in any predefined sequences.

T4 β-GT was previously employed for conjugating various modified glucose derivatives with 5hmC in DNA [31], [32]. Furthermore, some glucosyltransferase might catalyze the formation of GlcNAc-5hmU in some phages (e.g. *Mycobacteriophage Acadian*) [9]. Thus, T4 β-GT may be potentially useful for transferring modified Glc derivatives to 5hmU in ODNs, which may be utilized for introducing glucose derivatives with a bioorthogonal handle for labeling and assessing the distribution of 5hmU in DNA.

**Figure 4:**
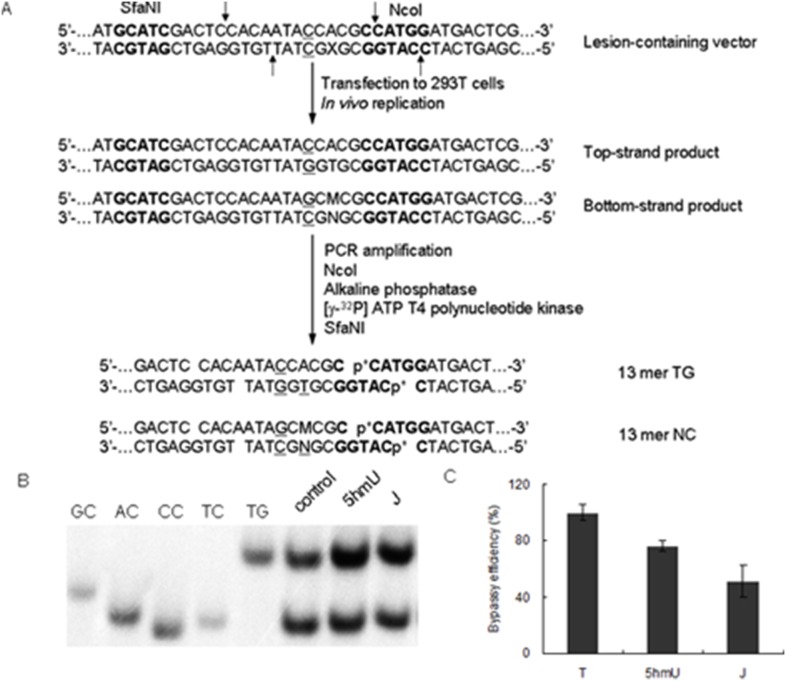
*In-vivo* replication studies of base J and 5hmU in HEK293T cells. (A) Experimental procedures for determining the effects of 5hmU and base J on DNA replication in cells. (B) Representative PAGE gel image showing the restriction fragments. (C) The bypass efficiencies of base J and 5hmU in HEK293T cells. The data represent the mean and standard deviation of results from three independent replication experiments.

Owing to the presence of a bulky glucose ring, base J blocks DNA transcription [10]; however, it remains unexplored how base J affects DNA replication. Thus, we investigated how base J perturbs DNA replication in mammalian cells using our recently developed shuttle vector method [22]. We first constructed the 5hmU- and base J-containing double-stranded plasmids, and transfected them into HEK293T cells. After cellular DNA replication, the progeny plasmids were isolated and amplified with PCR. The PCR products were subsequently digested with restriction enzymes, and the resulting restriction fragments were analyzed by PAGE and LC-MS/MS for product identification and quantification (Figure 4A). In this respect, the negative-ion ESI-MS and MS/MS data identified the [M–3H]^3−^ ions of non-mutagenic products d(CATGGCGTGCTAT) ([M–3H]^3−^, *m/z* 1320.4) and d(CATGGCGTGGTAT) ([M–3H]^3−^, *m/z* 1333.1) (Figure S4 & S5 in File S1). The ion of *m/z* 1315.7 observed in Figure S4 in File S1 could be attributed to the [M–3H]^3−^ ion of the bottom-strand sequence with T→C mutation, i.e., d(CATGGCGCGCTAT), or the [M+K–4H]^3−^ ion of the top-strand sequence, i.e., d(CACAATAGCACGC). The MS/MS showed that the ion of *m/z* 1315.7 arises from the top-strand sequence d(CACAATAGCACGC) (Figure S6 in File S1). Our LC-MS/MS results also revealed the absence of T→A or T→G mutation products, i.e., d(CATGGCGAGCTAT) or d(CATGGCGGGCTAT), in the restriction digestion mixture. Taken together, only the non-mutagenic sequences, i.e., d(CATGGCGTGGTAT) and d(CATGGCGTGCTAT) could be detected in the digestion mixtures for samples arising from the *in vivo* replication of base J- and 5hmU-containing substrates, which is in line with what we found from PAGE analysis (Figure 4B).

The bypass efficiencies were calculated from the ratio of the restriction products from the base J- or 5hmU-containing strand over that of the lesion-free strand (Figure 4B). Our results revealed that the bypass efficiencies for 5hmU and base J are approximately 80% and 52%, respectively (Figure 4C). Thus, 5hmU and base J constitute modest and moderate blocks to DNA replication machinery in human cells, respectively. Our result also revealed the absence of mutation introduced by base J during replication. Loss of base J could lead to cell death in *Leishmania* [10]. After DNA replication, some thymine residues in the nascent DNA strands must undergo rapid conversion to yield base J so that the levels of the modified base can be maintained. The high fidelity in replication across base J and its intermediate 5hmU is essential for the maintenance of base J levels during cell division.

## Conclusions

In summary, we reported an enzymatic method for the facile synthesis of base J-containing ODNs. The method also holds potential for incorporating modified glucose derivatives to 5hmU in ODNs. In addition, we demonstrated that, in contrast to its strong inhibitory effects on DNA transcription, base J only moderately impedes DNA replication in human cells.

## Supporting Information

File S1 Contains Figures S1–S6.
(DOC)
