# Supplementary material for: Facile Enzymatic Synthesis of Base J-Containing Oligodeoxyribonucleotides and an Analysis of the Impact of Base J on DNA Replication in Cells
Source: PLoS One. 2014 Jul 25;9(7):e103335. doi: 10.1371/journal.pone.0103335 (PMC4111573; doi:10.1371/journal.pone.0103335)
Supplement: File S1 — Contains Figures S1–S6. (DOC) [file pone.0103335.s001.doc]

**Supporting information for**

“Facile Enzymatic Synthesis of Base J-containing Oligodeoxyribonucleotides and an Analysis of the Impact of Base J on DNA Replication in Cells”

by Debin Ji and Yinsheng Wang

Figure S1 Denaturing polyacrylamide gel electrophoresis (PAGE) for monitoring the reaction mixtures of ODNs treated with T4 β-GT. Lanes 1 and 2 are the standard 12mer 5hmU- and base J-containing ODNs, respectively. Lanes 3-6 are double-stranded ODNs with 5hmU being paired with A, T, C and G, respectively. Lane 7 is single-stranded 12mer 5hmU-containing ODN after treatment with T4 β-GT.

Figure S2. Experimental procedures for the construction of 5hmU- and base J-containing duplex vectors. (A) A schematic diagram showing the procedures for the preparation of double-stranded plasmid harboring a site-specifically incorporated 5hmU or base J. (B) Enzymatic digestion and ligation for the insertion of 5hmU- or J-carrying ODN into gapped pTGFP-Hha10 vector. “X” represents T, 5hmU, or base J, and the C:C mismatch site is underlined. The restriction recognition sites are highlighted in bold, and cleavage sites are indicated by arrows.

Figure S3. Determination of the bypass efficiencies and mutation frequencies of 5hmU or base J using LC/MS. Restriction digestion of PCR products of progeny genome arising from *in-vivo* replication of 5hmU- and base J-bearing genomes in mammalian cells. The PCR fragments were treated simultaneously with NcoI, shrimp alkaline phosphatase and SfaNI. The ODN mixture was subjected to LC-MS/MS analysis. The restriction recognition sites are highlighted in bold, and cleavage sites are indicated by arrows.

Figure S4. Negative-ion ESI-MS revealing the digested ODN mixture from replication of base J- containing plasmids. The [M – 3H]^3-^ and the corresponding Na^+^/K^+^ adduct ions for d(CACAATAGCACGC), d(CATGGCGTGCTAT), and d(CATGGCGTGGTAT) are designated in normal, bold, and italic fonts, respectively.

Figure S5. LC-MS/MS for monitoring the restriction fragments of d(CATGGCGTGCTAT) and d(CATGGCGTGCTAT). Shown in (A) and (B) are the MS/MS of the [M – 3H]^3-^ ions (*m/z* 1320.4 and 1334.1) of these two ODNs.

Figure S6. MS/MS of the ion of *m/z* 1315.7 found in Figure S5, showing that the of *m/z* 1315.7 ion is from the [M – 4H + K]^3-^ ion of d(CACAATAGCACGC).
